# Targeted and direct intracellular delivery of native DNAzymes enables highly specific gene silencing

**DOI:** 10.1039/d0sc03974h

**Published:** 2020-08-07

**Authors:** Xia Li, Fang Yang, Wenjiao Zhou, Ruo Yuan, Yun Xiang

**Affiliations:** Key Laboratory of Luminescence Analysis and Molecular Sensing, Ministry of Education, School of Chemistry and Chemical Engineering, Southwest University Chongqing 400715 P. R. China yunatswu@swu.edu.cn

## Abstract

DNAzymes exhibit high potential as gene silencing agents for therapeutic applications. Such purposes, however, are significantly challenged by the targeted and successful delivery of unmodified DNAzymes into cells with minimal side effects. Here, we set out to formulate and demonstrate a new stimuli-responsive and constrained aptamer/DNAzyme (Apt/Dz) catenane nanostructure for highly specific gene silencing. The rational design of the Apt/Dz catenane nanostructure with the respective integration of the aptamer sequence and the completely closed catenane format enables both the targeted capability and significantly improved nuclease resistance, facilitating the stable and targeted delivery of unmodified Dz into cancer cells. Moreover, the Dz enzymatic activity in the constrained structure can only be conditionally regulated by the specific intracellular mRNA sequences to silence the target gene with highly reduced side effects. Results show that the Apt/Dz catenane nanostructure can effectively inhibit the expression of the target gene and the proliferation of cancer cells with high specificity.

## Abstract 2

The stimuli-responsive and constrained aptamer/DNAzyme catenane nanostructure enables the targeted delivery of native DNAzymes for highly specific gene silencing.

## Graphical abstract

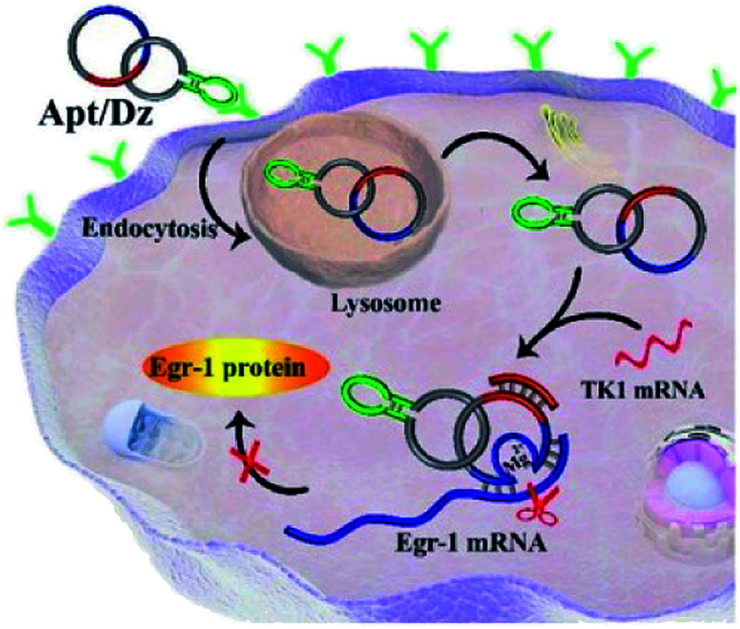

## Introduction

Gene silencing involves targeting specific mRNA sequences in cells to inhibit gene expression before translation.^1,2^ Because the inhibition or regulation of the expression of a certain gene *via* gene silencing can attenuate the invasion, proliferation and migration of cancer cells,^3,4^ gene silencing has been increasingly investigated as a novel therapeutic strategy for various cancers and diseases. The agents commonly used for gene silencing include small interfering RNAs, DNAzymes, ribozymes, microRNAs and antisense oligonucleotides, and DNAzymes have been shown to be a promising class of nucleic acid-based gene silencing agents because of their high specificity and substrate flexibility.^5^ DNAzymes are *in vitro*-selected catalytic nucleic acids that can catalyze a variety of reactions including DNA/RNA ligation,^6^ nucleic acid cleavage,^7–10^ Diels–Alder reaction^11^ and DNA phosphorylation.^12,13^ Of particular interest is that DNAzymes can selectively bind their substrate mRNA sequences, exhibiting catalytic activity comparable to that of protein enzymes to cleave these mRNAs during the translational repression of the target genes. Besides, DNAzymes show improved stability over other gene silencing agents, avoid the use of proteins for catalytic activity and are non-toxic and non-immunogenic, making them particularly suitable silencers at the molecular mRNA level.

Despite these advantages, their instability in biological media, targeted delivery and low efficiency of cellular uptake remain the major challenges for employing DNAzymes as potent therapeutics.^14–17^ The hydrophobic nature of cell membranes prevents the highly hydrophilic DNAzymes from crossing the membranes to enter cells. Efforts using cationic molecules,^18^ nanomaterials^19–24^ and liposomes^25,26^ as carriers to ferry DNAzymes into cells have been extensively reported. Nevertheless, these carriers required complicated synthetic approaches and may show potential cytotoxicity to the cells. On the other hand, the modification of DNAzymes at the 3′-termini with 3′–3′ inverted nucleotides^27^ and the use of phosphorothioate linkages^28^ as well as the integration of locked nucleic acids^29,30^ could indeed improve the nuclease resistance of DNAzymes. These nucleotide changes to DNAzymes, however, could result in the issues of cytotoxicity and the reduction of their catalytic potency.^31,32^ Apart from their delivery and stability, the target capability and side effects of DNAzymes for therapeutic purposes have rarely been addressed so far.

In this regard, we report herein a constrained and stimuli-responsive aptamer/DNAzyme (Apt/Dz) catenane nanostructure for targeted delivery of DNAzymes for highly specific gene silencing. The aptamer segment in the Apt/Dz that binds the specific receptors over-expressed on the tumor cell surfaces can facilitate the targeted delivery of the nanostructure inside the cells. Owing to the completely closed ring structure, such Apt/Dz catenane shows significantly improved stability against nuclease digestion, which makes it last chemically longer in lysosomes and increases its chances of release. Importantly, the controlled activation of the constrained DNAzyme activity regulated by the specific intracellular mRNA sequences can minimize the side effects of the DNAzyme for gene silencing.

## Results and discussion

**Scheme 1:**
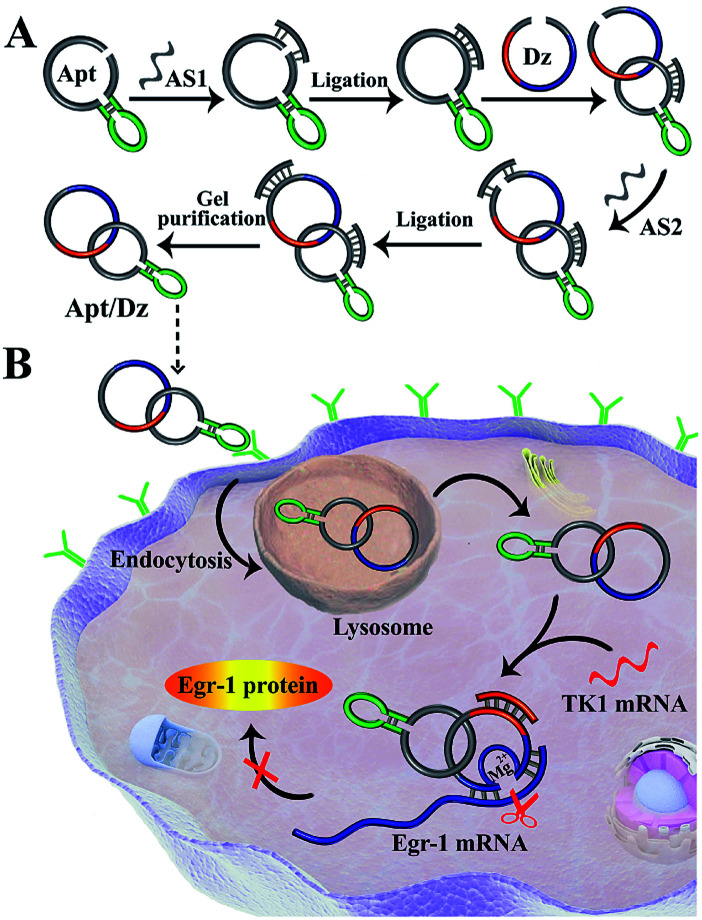
Illustration of (A) the preparation of the Apt/Dz constrained catenane nanostructure, and (B) the targeted delivery of the Apt/Dz nanostructure into cancer cells for gene silencing regulated by the specific TK1 mRNA sequences.

The synthesis of the thymidine kinase 1 (TK1) mRNA-responsive Apt/Dz constrained catenane nanostructure and its application for gene silencing is illustrated in Scheme 1. For a proof-of-concept demonstration, the 10–23 DNAzyme, which represents the most widely investigated DNAzyme for RNA cleavage,^33^ is selected as the therapeutic sequence to target the early growth response-1 (Egr-1) gene that is critical for tumor angiogenesis, growth and neovascularization.^34,35^ In addition, MCF-7 tumor cells with elevated TK1 mRNA concentration inside the cells^36,37^ are used as the target cells, and the mucin1 (MUC1) protein expressed on the MCF-7 cell surface is exploited as the receptor for targeted delivery of the catenane nanostructure into the cells. As shown in Scheme 1A, the MUC1 aptamer (the green segment)-containing sequence is firstly circularized by T4 DNA ligase with the assistance of a ligation probe, followed by its hybridization with the 10–23 DNAzyme (the blue region)-containing strand and further ligation of the nicks to prepare the Apt/Dz constrained nanostructure upon gel purification. In the design of such a constrained nanostructure, the DNAzyme is initially de-activated by partially complementing with the aptamer-containing sequence and can only be activated upon hybridization between the specific TK1 mRNA stimulus and the corresponding recognition sequence (the red segment). Once incubated with the target cells, the Apt/Dz nanostructures can be readily delivered into the cells *via* an endocytic pathway through their binding to the MUC1 proteins on the surfaces. The TK1 mRNA sequences inside the cells then hybridize with the nanostructures and cause their structure switching to activate the DNAzymes, which recognize and cleave the target Egr-1 gene to realize the gene silencing function.

**Fig. 1:**
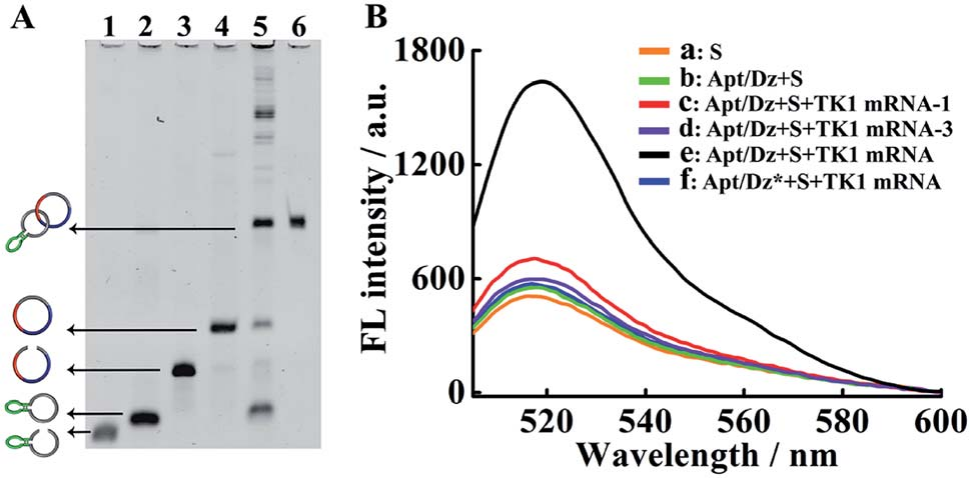
(A) Denaturing PAGE (8%) characterization for the preparation of Apt/Dz. Lane 1: Apt; lane 2: Apt + AS1 + ligase; lane 3: Dz; lane 4: Dz + AS2 + ligase; lane 5: circularized Apt + Dz + AS2 + ligase; lane 6: purified Apt/Dz. The concentrations of Apt, Dz, AS1, AS2 and ligase were 500 nM, 500 nM, 2 μM, 2 μM, and 40 U, respectively. Exonuclease I/III was used to degrade the un-ligated ssDNA. (B) Fluorescence assays for TK1 mRNA-induced activation of Dz activity in Apt/Dz: (a) S; (b) Apt/Dz + S; (c) Apt/Dz + S + TK1 mRNA-1; (d) Apt/Dz + S + TK1 mRNA-3; (e) Apt/Dz + S + TK1 mRNA; (f) Apt/Dz* + S + TK1 mRNA. The concentrations of S, Apt/Dz, Apt/Dz*, TK1 mRNA-1 and TK1 mRNA-3 were all 100 nM and the concentrations of the TK1 mRNAs were 50 nM. Solutions were incubated for 2 h in the presence of 10 mM Mg^2+^, followed by fluorescence measurements.

The stepwise preparation of the Apt/Dz constrained catenane nanostructure was first verified by denaturing polyacrylamide gel electrophoresis (PAGE). As can be seen in Fig. 1A, the respective circularization of the Apt and Dz sequences with the assistance probes (AS1 and AS2) and DNA ligase, followed by subsequent exonuclease I/III treatment to degrade the un-ligated ssDNA, leads to apparently reduced electrophoretic mobility against their linear counterparts (lane 2/4 *vs.* lane 1/3). These comparisons indicate that the two linear sequences of Apt and Dz can be successfully circularized as expected. The hybridization of the linear Dz with the circularized Apt, followed by further circularization of Dz, therefore results in the formation of a band (lane 5) with highly reduced mobility against either the circularized Apt (lane 2) or Dz (lane 4), suggesting the preparation of the Apt/Dz constrained catenane nanostructure. In addition, gel purification was performed to obtain the Apt/Dz nanostructure (lane 6). The specific TK1 mRNA-triggered recovery of Dz activity of the Apt/Dz nanostructure was also investigated by fluorescence spectroscopy using the fluorophore (FAM)/quencher (Dabcyl) pair-labeled RNA substrate sequence (S, 5′-TCG T-(Dabcyl)-CC AGG rArUG GCC GCG G-(FAM)-3′) that can be cleaved by the active Dz. According to Fig. 1B, substrate S alone in buffer shows a low fluorescence emission (curve a), due to the quenching of the FAM fluorescence by the Dabcyl quencher. The mixture of S and Apt/Dz has no significant influence on the fluorescence intensity because of the inhibition of the Dz activity in the constrained structure format (curve b *vs.* a). In addition, the incubation of the Apt/Dz catenane and S with one (TK1 mRNA-1, curve c) or three (TK1 mRNA-3, curve d)-base mismatched control sequences against TK1 mRNA exhibits similar fluorescence intensities to that of the mixture of Apt/Dz and S, while the incubation of such a mixture with TK1 mRNA leads to a drastically enhanced fluorescence (curve e) that can be ascribed to the cleavage of the substrate sequences by the active Dz restored upon specific binding between TK1 mRNA and Apt/Dz. Besides, in the presence of the specific TK1 mRNA sequences, the replacement of the binding arms of Dz with non-complementary regions (Dz*) to S also shows a comparable fluorescence (curve f) to that of the mixture of S and Apt/Dz (curve b). These comparisons clearly demonstrate that the activation of the Dz activity of Apt/Dz is highly dependent upon the specific TK1 mRNA trigger sequences.

**Fig. 2:**
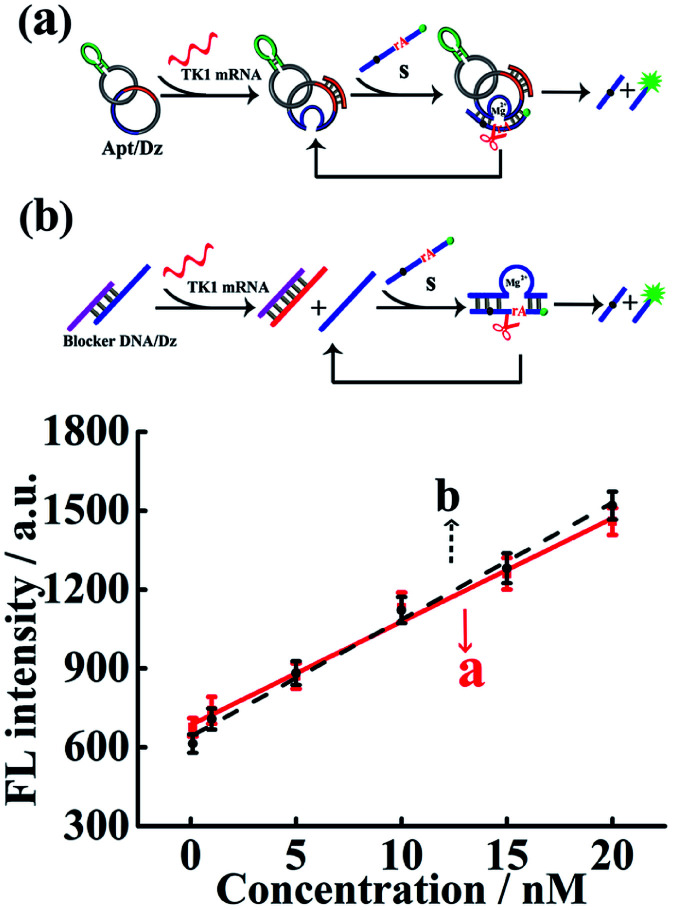
The comparison of Dz activity in the Apt/Dz catenane nanostructure (a) and the blocker DNA/Dz duplex (b) for TK1 mRNA detection at concentrations of 0, 100 pM, 1 nM, 5 nM, 10 nM, 15 nM and 20 nM. The TK1 mRNA was incubated with the Apt/Dz (100 nM) or the blocker DNA/Dz duplex (100 nM) for 2 h at 37 °C, followed by fluorescence measurements.

The Dz activity in the Apt/Dz catenane structure (Fig. 2a) was investigated and compared with that of the unmodified DNAzyme (generated by the TK1 mRNA-induced strand displacement of the blocker DNA/Dz duplex in Fig. 2b) *via* the detection of the TK1 mRNA sequence. As shown in Fig. 2, both Apt/Dz catenane (*F* = 39.3*c* + 684.7) and the blocker DNA/Dz (*F* = 44.4*c* + 640.2) display linear responses to the TK1 mRNA sequence in the range of 0.1–100 nM with the corresponding detection limits of 27 pM and 18 pM, respectively, based on the 3*σ* calculation. And, the *k* values are 39.3 and 44.4, respectively, for the Apt/Dz catenane and blocker DNA/Dz, indicating that the activity of Dz in the Apt/Dz catenane is not significantly affected upon being integrated into the nanostructure.

**Fig. 3:**
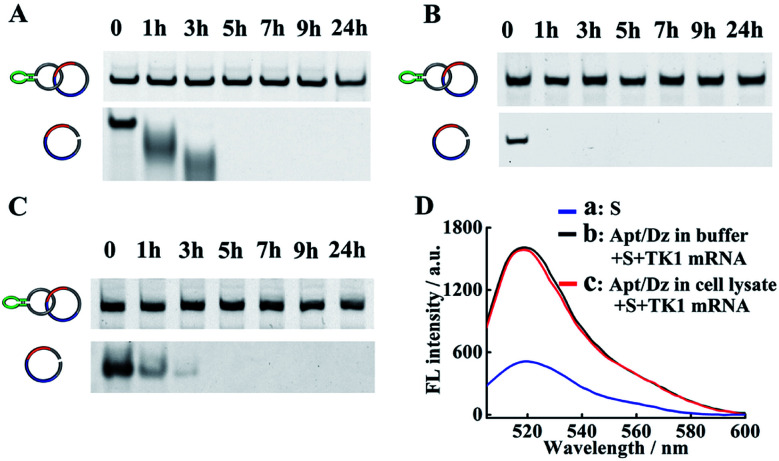
Stability analyses of the Apt/Dz catenane and the linear Dz incubated with (A) 10% FBS, (B) exonuclease I (0.5 U L^−1^) and (C) cell lysate of MCF-7 cells for different time frames by 6% native PAGE. (D) The catalytic activity assay of Apt/Dz: (a) S, (b) Apt/Dz incubated with buffer and (c) Apt/Dz incubated with cell lysate. Apt/Dz was incubated with cell lysate for 5 h, followed by being heated to 90 °C for 15 min and cooling down to 25 °C. TK1 mRNA and S were then added and incubated for 1.5 h at 37 °C before fluorescence measurement. The concentrations of S, Apt/Dz, TK1 mRNA and Mg^2+^ were 100 nM, 100 nM, 50 nM and 10 mM, respectively.

The successful delivery of Dz into cells for gene silencing is highly dependent on its resistance to nuclease attack, and previous findings have shown that circularized DNA exhibited significantly improved stability under biological conditions than the linear ones.^38^ To evaluate the stability of our Apt/Dz catenane, the nanostructure was, respectively, incubated with 10% FBS in DMEM, exonuclease I and the cell lysate of MCF-7 cells to mimic the physiological environment for different time frames by using the linear Dz as the control sequence, followed by native PAGE analyses. As displayed in Fig. 3A–C, significant degradation of the linear Dz by exonuclease I, or FBS or the cell lysate occurs within as short as 1 h with the disappearance of the band corresponding to the linear Dz. In contrast, the Apt/Dz catenane, however, is essentially stable up to 24 h in the incubation media, without apparent loss of the band intensity. The high stability of the Apt/Dz catenane in 10% FBS, exonuclease I and the cell lysate can be basically assigned to the fact that the elimination of the 3′ termini in the circularized sequences can restrict the access of the enzymes to its susceptible binding sites.^39,40^ The catalytic activity of Apt/Dz after being incubated with MCF-7 cell lysate was also evaluated against the untreated one. After the incubation of the Apt/Dz with the cell lysate for 5 h, the mixture was heated at 90 °C for 15 min to inactivate the enzymes in the lysate, followed by cooling down to 25 °C. Then, TK1 mRNA (50 nM) and S (100 nM) were added and incubated for 1.5 h before fluorescence measurement. According to Fig. 3D, only a very minor decrease in fluorescence intensity is observed for the Apt/Dz incubated with the cell lysate against the one incubated with buffer, confirming the maintenance of the catalytic activity of the Apt/Dz even upon cell lysate treatment. Such a stability therefore endows the Apt/Dz catenane with a significantly improved enzyme resistance to escape from lysosomes and to function inside cells for effective gene silencing.

**Fig. 4:**
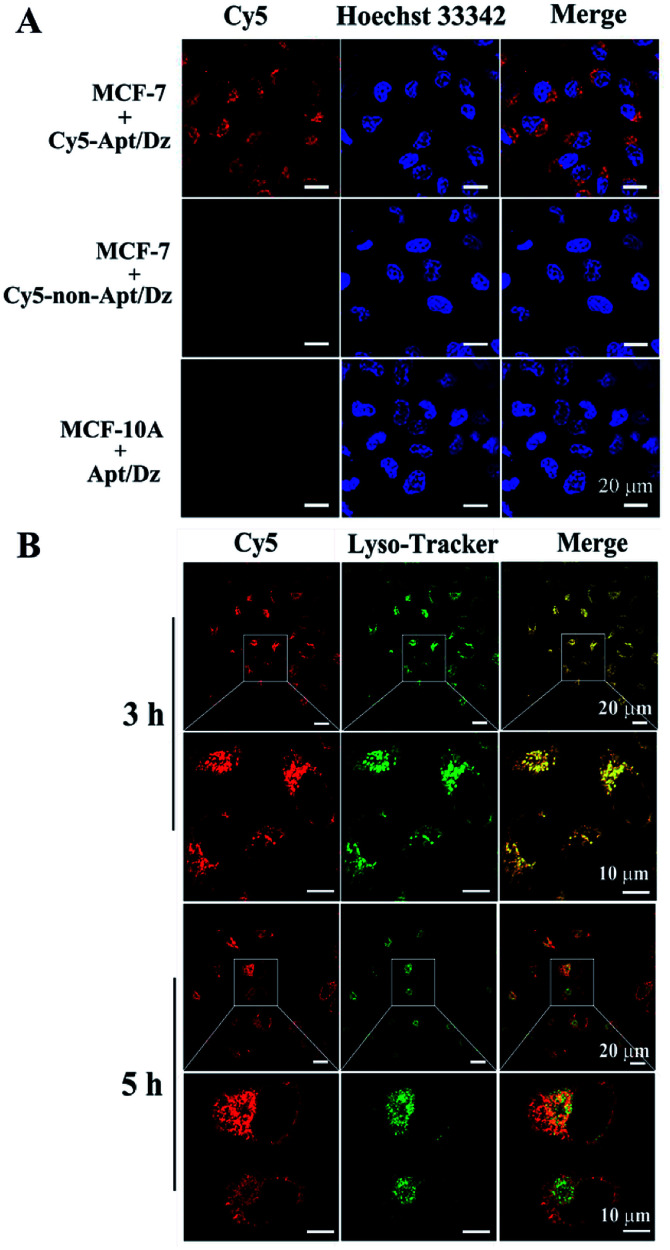
(A) MCF-7 and MCF-10A cells cultured with the Cy5-Apt (or non-Apt)/Dz catenanes and (B) subcellular localization analysis of MCF-7 cells cultured with the Cy5-Apt/Dz catenanes. The lysosomes and nuclei were, respectively, stained by Lysotracker (green) and Hoechst 33342 (blue).

After verifying its stability, the targeted delivery of the aptamer integrated Apt/Dz catenane into tumor cells was examined by using the confocal laser scanning microscope (CLSM) technique. In this respect, two different cell lines, the MCF-7 tumor cells (with over-expressed MUC1 on cell surfaces) and the normal MCF-10A cells (with highly reduced expression of MUC1),^41,42^ were cultured with the Cy5 fluorescent dye-labeled Apt/Dz catenane for 6 h. As can be seen in Fig. 4A, obvious red fluorescence from Cy5 is obtained inside the MCF-7 cells upon excitation because the over-expressed MUC1 molecules on the MCF-7 cell surfaces can bind the aptamer sequence in the Apt/Dz catenane to mediate its efficient delivery inside the cells in an endocytic pathway. In contrast, almost no fluorescence signals can be observed inside the MCF-10A cells, indicating that the Apt/Dz catenane is unable to be delivered inside MCF-10A cells mainly because of the lack of sufficient MUC1 on cell surfaces and the resistance and membrane permeability of the MCF-10A cells to the Apt/Dz catenane. In addition, the delivery of the Apt/Dz catenane into the cytosol, which is critical for gene silencing, was also investigated *via* subcellular localization assays. Fig. 4B shows that the Apt/Dz catenane nanostructures are mainly trapped in lysosomes based on the fluorescence overlap (yellow) between the fluorescence signals from the lysosomes stained by the LysoTracker dye (green) and the Cy5-Apt/Dz catenanes (red) after culturing the cells with the Cy5-Apt/Dz catenanes for 3 h. However, after 5 h of cell culture, the Cy5-Apt/Dz catenanes are mainly located in the cytosols with the separation of the green and red fluorescence signals, suggesting the successful escape of the Cy5-Apt/Dz catenanes from lysosomes with prolonged incubation time.

**Fig. 5:**
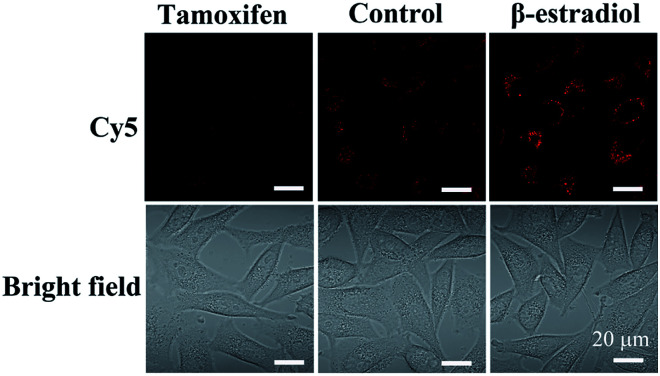
MCF-7 cells cultured with the Cy5-Apt/BHQ-2-Dz catenane for 6 h with/without the pretreatment with the TK1 mRNA expression regulation drugs.

Moreover, to demonstrate the structure switching of the nanostructure triggered by the TK1 mRNA inside the cells, the Cy5-Apt/Dz-BHQ-2 catenanes were respectively cultured with MCF-7 cells pretreated with two drugs, tamoxifen (down-regulation) and β-estradiol (up-regulation), which can regulate the expression of TK1 mRNA.^43–45^ According to Fig. 5, clear red fluorescence can be seen in MCF-7 cells without the pretreatment with the drugs while those pretreated with the tamoxifen/β-estradiol drugs exhibit reduced/intensified red fluorescence because of the variation of the expression level of TK1 mRNA caused by the two drugs. This observation reveals that the TK1 mRNA inside the cells can indeed cause a structure switching of the Apt/Dz catenane.

**Fig. 6:**
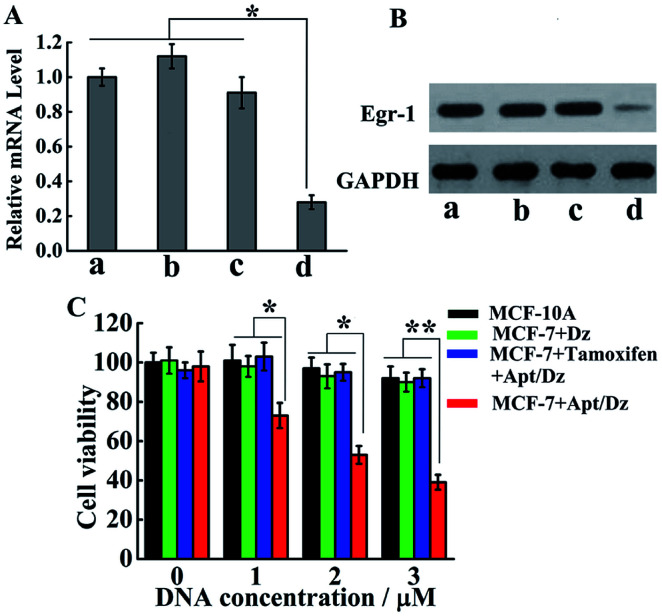
(A) The qRT-PCR and (B) western blot analyses of the expression levels of the Egr-1 mRNA and Egr-1 protein, respectively, in the untreated MCF-7 cells (a) and MCF-7 cells treated with non-Apt/Dz ((b) 3 μM), Apt/Dz* ((c) 3 μM) and Apt/Dz ((d) 3 μM). (C) MTT cell proliferation assays for the MCF-7 (with/without the pretreatment with the tamoxifen drug) and MCF-10A cells after 48 h incubation with various concentrations of Apt/Dz catenane and Dz. Error bars: SD, *n* = 3. *p < 0.05, **p < 0.01.

With the demonstration of the successful delivery of the Apt/Dz catenane into the target cells, the mRNA expression inhibition effect of the Apt/Dz catenane on the Egr-1 gene was further evaluated. For this purpose, MCF-7 cells were cultured with Apt/Dz and the control catenanes, including Apt/Dz* (Dz with non-complementary Egr-1 gene binding arms) and non-Apt/Dz (without the aptamer region), for 48 h, followed by qRT-PCR quantification of the Egr-1 mRNA expression level. Fig. 6A shows that the MCF-7 cells cultured with the Apt/Dz* and non-Apt/Dz catenanes show no obvious variations in Egr-1 mRNA expression levels against the untreated cells, due to neither the binding of Dz* with the Egr-1 gene (in agreement with curve f in Fig. 1B) nor the delivery of the non-Apt/Dz into the cells (in agreement with Fig. 4A). In contrast, the culture of the Apt/Dz catenanes with the MCF-7 cells leads to about 73% inhibition of the Egr-1 mRNA expression compared with the untreated cells, revealing the expected gene silencing capability of the Apt/Dz catenane. Additionally, the downstream Egr-1 protein expression was also analyzed by western blot to further confirm the inhibition of the Egr-1 mRNA expression. As can be seen in Fig. 6B, MCF-7 cells treated with the Apt/Dz catenanes exhibit a significant decrease in Egr-1 protein expression in contrast to those untreated or Apt/Dz* and non-Apt/Dz-treated cells because of the inhibition of the Egr-1 mRNA in the cells by Apt/Dz catenanes, which conforms to the qRT-PCR analyses to verify the degradation of the Egr-1 mRNA. Considering the fact that the silencing of the mRNA can inhibit cell proliferation, the potential of the Apt/Dz nanostructure for the inhibition of the MCF-7 cell proliferation was investigated *via* cell viability studies. After culturing the Apt/Dz nanostructure at different concentrations with the target MCF-7 cells with/without the pretreatment with the tamoxifen drug and the control MCF-10A cells for 48 h at 37 °C, cell viability was evaluated by MTT assays. The results (Fig. 6C) show that the Apt/Dz nanostructure leads to increasing inhibition of the proliferation of the MCF-7 tumor cells without the pretreatment with tamoxifen when the concentration of the Apt/Dz is elevated from 1 to 3 μM, and 61% cell proliferation inhibition can be achieved at a concentration of 3 μM. However, the traditional Dz has an obviously limited inhibition efficiency (∼10%) even at a high concentration of 3 μM because of the aforementioned challenges in targeted delivery of the natural Dz into the MCF-7 cells as discussed previously. Meanwhile, the proliferation of MCF-7 tumor cells pretreated with tamoxifen (to inhibit the expression of the TK1 mRNA) and the MCF-10A cells is not obviously affected, suggesting its efficacy for cell proliferation inhibition in a highly selective fashion.

## Conclusions

To summarize, we have demonstrated the preparation of the constrained Apt/Dz catenane nanostructure and its application for highly specific gene silencing by the targeted delivery and the intracellular mRNA stimuli-triggered restoration of the Dz activity. The constructed DNA nanostructure exhibits the advantages of targeted capability, improved stability, non-toxicity and minimized side effects, which are critical to the use of Dz as therapeutic drug. By tailoring the aptamer sequence in the nanostructure that can target different cell type receptors, the design of simple and robust Dz drugs for inhibiting the proliferation of various cancer cells can therefore be envisioned.

## Experimental

### Materials

The Rapid DNA Ligation Kit, exonuclease I/III (Exo I/III), Lipofectamine 3000, and LysoTracker™ Green DND-26 were ordered from Thermo Scientific (Waltham, MA, USA). Urea and *N*,*N*,*N*′,*N*′-tetramethylethylenediamine (TEMED) were provided by Aladdin (Shanghai, China). MCF-7 and MCF-10A cells were purchased from the cell bank of the type culture collection of the Chinese Academy of Sciences (Shanghai, China). 3-[4,5-Dimethylthiazole-2-yl]-2,5-diphenyltetrazolium bromide (MTT) reagents and Hoechst 33342 were purchased from Beyotime (Haimen, China). NaCl, MgCl_2_, Tris, ammonium persulfate, acrylamide/bis-acrylamide (39 : 1, 30%), formamide, 10× TBST buffer and HPLC-purified oligonucleotides were all bought from Sangon Biotech Co., Ltd (Shanghai, China). The sequences of the oligonucleotides were as follows: Apt (or Cy5-Apt): 5′-Phos-GCA GTT GAT CCT TTG GAT ACC CTG GTT TTT GAC ACT CGC TAT AGT-(Cy5)-CCA GGA ATT ATG TGT CTA A-3′; AS1: 5′-AGG ATC AAC TGC TTA GAC ACA TAA-3′; Dz (or BHQ-2-Dz): 5′-Phos-CAA TCT AAG TAC CCG CGG CCA GGC TAG CTA CAA CGA CCT-(BHQ-2)-GGA CGA TAG CGA GTG TCT TTG GCA TAC TTG ATC AAA AAA ATA GCG AGT GTC AA-3′; Dz*: 5′-Phos-CAA TCT AAG TAC CCG CCG ACA GGC TAG CTA CAA CGA TCT CGA CGA TAG CGA GTG TCT TTG GCA TAC TTG ATC AAA AAA ATA GCG AGT GTC AA-3′; AS2: 5′-GTA CTT AGA TTG TTG ACA CTC GCT-3′; non-Apt: 5′-Phos-CAC TAC TTA CAG ATA GAC TGC AGC ATT TTT GAC ACT CGC TAT AGT CCA GGA ATT ATG TGT CTA A-3′; AS3: 5′-CTG TAA GTA GTG TTA GAC ACA TAA-3′; substrate strand (S): 5′-TCG T-(Dabcyl)-CC AGG rArUG GCC GCG G-(FAM)-3′; TK1 mRNA: 5′-UGA UCA AGU AUG CCA AAG ACA CUC GCU A-3′; TK1 mRNA-1: 5′-UGA UCA AGU AUG CCA AAC ACA CUC GCU A-3′; TK1 mRNA-3: 5′-UGA UCU AGU AUG CCA AAC ACA UUC GCU A-3′; blocker strand: 5′-CAC TCG CTA TCG TCC AGG -3′.

### Preparation of Apt/Dz

Firstly, the mixture of Apt (1 μM) and AS1 (4 μM) was annealed at 90 °C for 5 min and then immediately cooled to 25 °C. The resulting solution was allowed to react for 40 min at 25 °C, followed by the addition of the T4 quick ligase and further incubation at 25 °C for 5 h to prepare the circularized Apt. Exo I/Exo III was then added to the mixture and incubated for 30 min to digest the linear DNA, followed by deactivating Exo I/Exo III at 80 °C for 15 min. After that, Dz (1 μM) was added and incubated with the solution for 1 h, followed by the addition of AS2 (4 μM) and further incubation for another 1 h. This was followed by the addition of the T4 quick ligase and incubation at 25 °C for 5 h to obtain the Apt/Dz catenane. Gel purification of Apt/Dz was performed using a UNIQ-10 Spin Column DNA Gel Extraction Kit (Sangon Biotech, China) as follows: (1) gels containing the assembled Apt/Dz were excised carefully and immersed in the diffusion buffer; (2) the centrifuge tubes containing the smashed gels were placed in a 55 °C water bath for 2 h to promote the diffusion of DNA into the solution; (3) the mixture was centrifuged at 10 000 rpm for 10 min and the supernatant solution was collected; (4) binding buffer II and isopropanol were added into the supernatant and the resulting solutions were all transferred to the adsorption column, which was allowed to adsorb for 2 min and centrifuged at 8000 rpm for 30 s; (5) after removing the liquid, elution buffer was added into the adsorption column; (6) the adsorption column was centrifuged again to collect Apt/Dz.

### Denaturing and native polyacrylamide gel electrophoresis (PAGE)

The 6× loading buffer (2 μL) was added into each of the reaction samples (10 μL) and these solution mixtures were transferred into the freshly prepared gels. The denaturing PAGE (8%, 7 M urea) was run in 1× TBE at 120 V for 1.5 h and native PAGE (6%) was run in 1× TBE at 100 V for 1 h. After that, the gels were stained by Gel Red and imaged by the Bio-Rad imaging system (Hercules, CA, USA).

### Fluorescence assay of TK1 mRNA-triggered restoration of Dz activity for the cleavage of the mRNA substrate

The mixture of Apt/Dz (100 nM) and the mRNA substrate sequence S (100 nM) was incubated without/with the TK1 mRNA, TK1 mRNA-1, and TK1 mRNA-3, respectively, for 2 h at 37 °C in 5 mM Tris buffer (pH 7.5, 150 mM NaCl, 10 mM MgCl_2_). The fluorescent responses of the solutions with an excitation wavelength of 490 nm were then recorded using a Shimadzu RF-6000 spectrofluorophotometer (Tokyo, Japan).

### Cell culture

MCF-7 and MCF-10A cells were separately cultured in Dulbecco's Modified Eagle Medium (DMEM) and Roswell Park Memorial Institute 1640 (RPMI-1640) in a 5% CO_2_ humidified chamber at 37 °C with the culture medium containing penicillin (100 U mL^−1^), streptomycin (100 mg mL^−1^) and 10% fetal bovine serum (FBS).

### Cellular uptake of Apt/Dz, subcellular localization assay and TK1 mRNA-dependent structure switching of Apt/Dz

For intracellular uptake studies, cells were firstly seeded onto a 35 mm^2^ confocal dish and cultured in growth media to reach approximately 80% confluence. After rinsing twice with PBS, cells were incubated with the probes for 6 h. Next, cells were further rinsed using PBS and stained by 1 μg mL^−1^ Hoechst 33342 for 10 min before imaging on an LSM 900 confocal microscope (Zeiss, Germany). For the subcellular localization assays, Cy5-Apt/Dz (100 nM) was introduced into the culture medium for the MCF-7 cells and cultured with the cells for 3 and 5 h, respectively, followed by a thorough washing with PBS and staining the cells with LysoTracker™ Green (50 nM) for 30 min before CLSM imaging. For the TK1 mRNA-dependent structure switching of Apt/Dz, three groups of MCF-7 cells were separately cultured with PBS (control), tamoxifen (10^−6^ M) and β-estradiol (10^−8^ M) for 48 h. The fresh culture medium containing Cy5-Apt/Dz-BHQ-2 (1 μM) was then introduced and cultured with the cells for 6 h before CLSM imaging.

### The qRT-PCR quantification of the Egr-1 mRNA and the western blot assay

MCF-7 cells were cultured in a 6-well plate to reach approximately 80% confluence. Subsequently, the cell medium was quickly replaced by a fresh medium containing 3 μM non-Apt/Dz, Apt/Dz* or Apt/Dz, followed by 12 h of incubation at 37 °C. After replacing the culture medium, cells were further cultured in fresh medium for another 36 h, followed by PBS washing. Subsequent extraction and reverse-transcription of the RNA samples from the MCF-7 cells were carried out using the Trizol Reagent (Invitrogen, USA) and the Prime Script™ II 1st strand cDNA Synthesis Kit (Takara, Japan), respectively. The qRT-PCR detection of the Egr-1 mRNA was performed on a CFX96™ Real-Time System using the Power SYBR Green PCR Master kit (ABI, USA) with the following primers: forward primer sequence (Egr-1): 5′-AAC AGT GGC AAC ACC TTG TG-3′; reverse primer sequence (Egr-1): 5′-ACT GGT AGC TGG TAT TGA GG-3′. For the western blot assay, the extraction of the proteins from the MCF-7 cells was carried out using the RIPA Lysis Buffer (Beyotime, Haimen, China) and the concentrations of the extracted protein were determined using the BCA Protein Assay Kit (Beyotime, Haimen, China). The extracted proteins were separated using 8% SDS-PAGE gel and carefully transferred to a 0.2 μm PVDF membrane, followed by the blocking treatment in 5% nonfat milk. The anti-Egr-1 antibody (1 : 1000, Abcam, UK) was then incubated with the membrane overnight. Subsequently, the horseradish peroxidase-conjugated goat anti-rabbit IgG (1 : 10 000, Beyotime, China) was added and incubated for 2 h, followed by washing the membrane with TBST and imaging it with a ChemiDoc XRS+ system (Bio-RAD). For comparison, the GAPDH antibody was used as the control.

### Cell proliferation assays

MCF-7 and MCF-10A cells were cultured in a 96-well plate to reach approximately 80% confluence. Subsequently, cell media were quickly removed and fresh media containing various concentrations of Apt/Dz were added and incubated for 12 h. After replacing the culture medium, cells were further incubated in fresh medium for another 36 h. The cells were then incubated with 5 mg mL^−1^ MTT at 37 °C for 4 h, followed by removing the supernatant. DMSO (100 μL) was added into each well for 15 min with shaking. Finally, the absorbance value at 490 nm was monitored using an RT 6000 microplate reader.

### Statistical analysis

Statistical analyses of data variances between groups were performed using Student's *t*-test in GraphPad Prism 5.0. *p < 0.05 and **p < 0.01 represented significant results.

## Conflicts of interest

The authors declare no conflict of interest.
